# Neurovascular Issues in Neurofibromatosis Type I: Focus on Intracranial Stenosis

**DOI:** 10.3390/life16020234

**Published:** 2026-02-01

**Authors:** Marialuisa Zedde, Rosario Pascarella

**Affiliations:** 1 Neurology Unit, Stroke Unit, Azienda Unità Sanitaria Locale-IRCCS di Reggio Emilia, Viale Risorgi-Mento 80, 42123 Reggio Emilia, Italy; 2 Neuroradiology Unit, Ospedale Santa Maria della Misericordia, AULSS 5 Polesana, 45100 Rovigo, Italy; rosario.pascarella@aulss5.veneto.it

**Keywords:** NF1, angiography, MRI, MRA, intracranial stenosis, moyamoya, ICA, DSA

## Abstract

**Background/Objectives:** Neurofibromatosis type 1 (NF1) is a genetic disorder characterized by various clinical manifestations, including significant neurovascular complications. This review aims to synthesize current knowledge regarding intracranial stenoses and associated vascular abnormalities in patients with NF1, emphasizing the differences between pediatric and adult populations. **Methods:** A narrative review was conducted, analyzing the existing literature on the epidemiology, clinical manifestations, and management of neurovascular issues related to NF1. Data were collected from a range of studies, including retrospective analyses and case series, focusing on the incidence and outcomes of intracranial vascular abnormalities. **Results:** The study found that intracranial vasculopathy affects between 0.4% and 6.4% of NF1 patients, with children experiencing higher rates of stenotic lesions. However, vascular issues in adults are less understood, with 3.5% of adult patients presenting vascular abnormalities. The review highlights a significant underdiagnosis of these conditions due to the lack of routine use of magnetic resonance angiography (MRA) in standard evaluations. The management of NF1-related vascular conditions, particularly in adults, remains poorly defined, particularly regarding the efficacy of antithrombotic therapies. **Conclusions:** The management of neurovascular complications in NF1 requires urgent attention, with a need for standardized screening protocols and further research to elucidate the natural history and optimal treatment strategies for these patients. Enhanced diagnostic practices, including routine neuroimaging, are essential to improve outcomes and reduce the risk of significant vascular events.

## 1. Introduction

Neurofibromatosis type 1 (NF1) is a genetic condition first identified in 1882 by German pathologist Friedrich Daniel von Recklinghausen [1]. For many years, the understanding of NF1 was primarily descriptive; however, advancements in genetics during the 20th century enhanced the comprehension of its hereditary nature. NF1 (MIM 162200) is an autosomal dominant disorder due to pathogenic mutations of the NF1 gene on chromosome 17, encoding the neurofibromin protein. Acting as a tumor suppressor, mutations in neurofibromin lead to the hallmark symptoms of NF1, including multiple neurofibromas, café-au-lait spots, and skeletal irregularities.

NF1 is the most prevalent neurocutaneous disorder worldwide, with an estimated incidence of 1 in 3000 [2]. Although NF1 follows an autosomal dominant inheritance pattern, approximately 50% of cases are due to de novo mutations and therefore do not have a familiar history [2]. This disease affects several organ systems with significant clinical variability both inter- and intra-familially, without a strong genotype–phenotype relation. Diagnosis relies primarily on the established clinical criteria first proposed by the National Institutes of Health (NIH) in 1987 and their subsequent updates. In fact, advancements in molecular genetics and a better understanding of the pathophysiology led to add to these criteria newer diagnostic markers, such as specific chromosomal abnormalities, to enhance diagnostic precision and facilitate early detection. NF1 is diagnosed clinically when two or more features are present, including more than six café-au-lait macules, skinfold freckling, Lisch nodules, characteristic bone lesions (e.g., sphenoid wing dysplasia), optic pathway gliomas, neurofibromas of the skin or deep nerves, and a first-degree relative with NF1 [3]. Despite the usefulness of these criteria, they often fail to identify NF1 in young children, with 46% of sporadic cases not meeting the criteria by the age of one [4]. The revised criteria from 2021 incorporate genetic testing for NF1 gene variants and choroidal anomalies (CAs), allowing for earlier and more accurate diagnoses [5,6,7,8].

Currently, over 3000 distinct pathogenic variants of the NF1 gene have been documented, predominantly consisting of loss-of-function mutations (including nonsense and missense mutations, deletions, insertions, frame-shifts, and translocations) [9].

Vascular abnormalities represent a significant complication for patients with NF1 [10]. Cerebrovascular issues, which encompass narrowed or ectatic vessels, stenoses, aneurysms, and moyamoya disease, occur in approximately 2.5% of pediatric NF1 patients [10]. In the adult population, the prevalence of vascular abnormalities is about 3.5%, with stroke and cerebral arterial stenosis being the most frequently encountered issues [11]. Additionally, the presence of arterial hypertension can affect the risk of vascular complications in NF1 patients, including stroke, The prevalence of arterial hypertension in NF1 has been reported as around 1% [12]. However, incidence of arterial hypertension increases with age [13]. The leading causes of hypertension are essential hypertension and secondary causes, such as pheochromocytoma, renal artery stenosis, and coarctation of the abdominal aorta [13]. While primary hypertension is common, secondary causes are more frequently observed in NF1 patients than in controls [14]. One of the limitations of the available papers assessing the prevalence of vascular abnormalities in NF1 patients is the different selection of the cohorts. In fact, in a cohort of 31 patients retrospectively collected from referrals to vascular surgery in a tertiary center, the prevalence of each vascular involvement is considerably higher than in patients selected by dedicated NF1 outpatient services, with the prevalence of renal artery stenosis being 41%, cerebral vasculopathy 19%, and abnormalities of the abdominal aorta 12% [15]. While neurovascular involvement is less common than systemic vascular involvement, it is not negligible and may be underestimated in some cases. Previous reports indicate that vascular abnormalities in NF1 are often asymptomatic [16,17], although this data predominantly stems from pediatric cohorts [16].

We propose a narrative review with illustrative examples of the main neurovascular issues in patients with NF1. This topic has not been systematically addressed in the literature, and the heterogeneity of the available data prevents addressing it with a systematic review. We thus performed a literature search using PubMed starting from January 2000 using the following terms: (neurofibromatosis type 1 [MeSH Terms]) AND (intracranial vascular disease [MeSH Terms]). From the 151 results, we excluded case reports and case series of less than 10 patients; among the remaining papers, we selected the papers reporting retrospective or prospective cohorts with retrievable data about the main topic of the review and narratively described the most meaningful ones.

The primary objective of this narrative review is to consolidate the available information on this topic, focusing on intracranial arterial involvement and its differential diagnosis, to raise awareness about this condition in both children and adult patients. In fact, the lack of screening guidelines at the diagnostic stage and the lack of management recommendations in the following steps are relevant gaps in the knowledge needed for clinical practice.

## 2. Neurovascular Issues in NF1

### 2.1. Epidemiology and Clinical Phenotypes

The estimated incidence of cerebral vasculopathy in individuals with Neurofibromatosis type 1 (NF1) ranges from 0.4% to 6.4% [13,18,19]. In children specifically, this incidence is estimated to be between 2% and 5% [16]. The manifestations of cerebral vasculopathy are varied, including stroke [20], moyamoya arteriopathy [21,22], vessel stenosis and occlusion [23,24], aneurysms [10,25], vessel ectasia, fistulas, and ruptures. A well-established association exists between vasculopathy, particularly moyamoya arteriopathy, and patients with NF1 who have undergone cranial radiotherapy [26]. Less common vascular complications include superficial siderosis [27]. Sobata et al. categorized neurofibromatosis type 1-associated cerebral vasculopathy into three groups: stenotic, aneurysmal, or a combination of both [23]. They observed that 89% of children typically exhibited stenotic lesions, while the remaining cases involved aneurysmal formations. Follow-up studies revealed that nearly all children had stenosis or occlusion of the intracranial arteries [10]. Moyamoya disease is recognized in NF1 patients and may also arise secondarily due to radiation-induced changes following treatment for head and neck cancers [28]. However, a large Finnish population-based study did not find a significant association between intracranial aneurysms and NF1 in individuals with aneurysmal subarachnoid hemorrhage [29].

### 2.2. Stroke

A significant vascular manifestation in NF1 is the occurrence of stroke. A contribution to this risk is provided by the presence of arterial hypertension in some NF1 patients [13]. Terry and colleagues, through a population-based case-control study using data from the US Nationwide Inpatient Sample, indicated that patients with NF1 tend to have a younger mean age at stroke onset, a higher likelihood of arterial hypertension in pediatric age, and an increased incidence of stroke compared to the general population, in particular hemorrhagic stroke [20]. The causes of arterial hypertension in these patients can include renal artery stenosis and, less commonly, underlying pheochromocytoma.

### 2.3. Molecular Mechanisms of NF1-Related Vasculopathy

NF1-associated vasculopathy impacts small, medium, and large vessels, with arterial involvement being the most prevalent, although venous involvement can also occur. The pathophysiology of this vasculopathy is believed to stem from the disruption of neurofibromin’s critical role in regulating vascular endothelium [30], smooth muscle cells [18,31,32], and bone marrow cells [33], along with macrophage dysfunction [34]. This disruption leads to neointimal hyperplasia, inflammation, and an exaggerated response to injury, including enhanced angiogenesis. Histological and ultrastructural evaluations of vessels in NF1 have revealed intimal thickening, smooth muscle nodules and proliferation, fibromuscular hyperplasia, Schwann cell hyperplasia, and neural proliferation within vessel walls [13].

However, the pathogenesis of NF1-related vasculopathy remains poorly understood, though it is likely associated with neurofibromin [35]. The NF1 gene is substantial, spanning approximately 335 kb on chromosome 17q11.2, and its protein product, neurofibromin, shares functional homology and sequence with a group of Ras-GTPase-activating proteins [36,37]. The inactivation of neurofibromin results in unregulated Ras activity, which subsequently activates several critical downstream signaling pathways, ultimately leading to increased cell proliferation [38]. Neurofibromin has been detected in the endothelial layers of cerebral and renal arteries, as well as in the aorta, in both rodent and bovine models. The dominant hypothesis suggests that the absence of neurofibromin expression in endothelial cells triggers excessive proliferation of vascular endothelial and/or smooth muscle cells [39]. Furthermore, neurofibromin is essential for preserving the integrity of the endothelial cell layer; mutations in this protein disrupt this integrity, resulting in unchecked proliferation of vascular smooth muscle cells. Notably, NF1-associated vasculopathy does not uniformly impact all arteries within an individual, even though all arteries share the same constitutional mutation of the NF1 gene. Possible mechanisms include a “second hit” mutation of the normal NF1 allele or somatic mutations at other loci that lead to vascular changes [18]. Additionally, the interaction between host and environmental factors may play a role in the selective development of vasculopathy.

Bajaj et al. explored the impact of neurofibromin loss on endothelial cells, finding that its absence leads to increased cell proliferation and entry into the cell cycle. Moreover, NF1-deficient cells exhibited abnormal morphogenesis, failing to branch normally in co-culture assays, resulting in fewer tubules and branches [30]. Early hypotheses regarding vasculopathy in NF1 suggested possible Schwann cell proliferation within arteries, as proposed by Salyer and Salyer based on morphological observations that indicated a resemblance to cellular areas of neurofibroma [40]. However, this hypothesis has lost traction, with evidence suggesting that other cell components, particularly smooth muscle, are more likely involved in the vasculopathy [18]. For example, research by Li and colleagues demonstrated increased proliferation and migration of vascular smooth muscle cells in cultures derived from Nf1 +/− mice and human patients with NF1. This increase was attributed to Ras-induced elevation of platelet-derived growth factor (PDGF) resulting from neurofibromin loss [31]. Thus, dysfunction of vascular smooth muscle cells due to neurofibromin deficiency may contribute to the development of vasculopathy.

Additionally, it has been proposed that NF1-associated vasculopathy arises from intimal proliferation resulting from macrophage dysfunction. Research by Stansfield and colleagues, which involved Nf1 +/− and wild-type mice, demonstrated that Ras-Erk-directed recruitment of macrophages was accompanied by increased macrophage proliferation, migration, adhesion, and neointimal formation following injury in neurofibromin-deficient mice compared to their wild-type counterparts [34]. Furthermore, Bessler and colleagues indicated that MCP-1, a chemokine that is both anchored to the endothelium and secreted, along with CCR2-positive macrophages, plays a crucial role in mediating neointima formation in the context of NF1 [41].

Histological and ultrastructural analyses of vessels in NF1 patients have shown thickened intima, smooth muscle nodules and proliferation, fibromuscular hyperplasia, Schwann cell hyperplasia, and neural proliferation within vessel walls in mouse models [13,42]. Neointimal thickening is observable in small-caliber vessels in the spinal cord, leptomeningeal vessels, and choroid plexus of individuals with NF1 [43]. Additionally, Nf1 deficiency has been shown to enhance neointimal formation in mice following injury [34].

Moreover, while NF1 is widely expressed, it is particularly abundant in neural crest (NC)-derived cell cultures, where it plays a crucial regulatory role in neural stem cell proliferation and precursor migration, with specific effects depending on the target cell type derived from different segments of the neural tube (cephalic, vagal, trunk, or sacral) [44].

A significant limitation in understanding the precise molecular mechanisms underlying neurovascular manifestations in NF1 is the absence of a reliable genotype–phenotype correlation. This is likely due to the underestimation of vascular issues in these patients and the frequent occurrence of de novo mutations. Multiple factors can influence phenotype, including age-dependent manifestations, allelic and non-allelic heterogeneity, the timing and nature of the second hit in specific cells, wild-type allele, modifier genes, environmental influences, and stochastic factors. The interaction of these factors determines a specific phenotype. NF1 clinical outcomes cannot be predicted by the type of NF1 PVs, and evidence for modifier genes has been obtained from large familial studies [45]. A study aimed at identifying a genotype–phenotype correlation, particularly regarding general vascular manifestations in children [46], identified Lys1423 missense variants as associated with cardiovascular abnormalities, mainly resembling Noonan-like features and pulmonary artery stenosis. Another mutation, the c.3826C>T (p.(Arg1276*) nonsense variant, correlates with cardiovascular disorders [47,48]. However, neurovascular issues are largely overlooked in NF1 research, contributing to the lack of reliable data on the underlying molecular mechanisms.

### 2.4. Children vs. Adults

The neurovascular manifestations of NF1 have been explored in both pediatric and adult populations, primarily through single-center case series and case reports. Some larger studies focusing on children have also been conducted and are relevant to this review, according to the methods described in the introduction section.

A 10-year retrospective study from the USA assessed the prevalence, clinical manifestations, management, and outcomes of cerebral vasculopathy in children with NF1 [49,50]. In this study, MRI was performed on 78% of the patients (312 out of 398), of whom 46% (143/312) also underwent intracranial MRA. The prevalence of cerebral vasculopathy was found to be 4.8% (15/312), with approximately half of these cases being asymptomatic at presentation. The manifestations included moyamoya arteriopathy (seven cases: five unilateral and two bilateral) and stenosis or occlusion of large intracranial arteries: eight cases: three had involvement of more than one major artery (internal carotid, middle cerebral, or posterior cerebral arteries, or ICA, MCA, and PCA, respectively); three had isolated supraclinoid ICA involvement (one occlusion and two stenoses); and two had isolated vertebral artery (VA) involvement (one occlusion and one stenosis)].

An Italian single-center case series identified multiple intracranial stenoses and occlusions in six patients (7.4%), with a median age of 17 years [51]. Another study utilized transcranial Doppler ultrasound as a screening tool for cerebral vasculopathy in 40 NF1 patients aged 5 to 19 years undergoing MRI [52]. Among these, 3 of the 40 patients (7.5%) exhibited vascular changes on MRI and/or MRA; 2 of these 3 patients were symptomatic. Conversely, 4 out of the 40 children showed cerebrovascular changes via transcranial Doppler, with three-quarters of them confirmed by MRA.

Recently, a large retrospective case series involving NF1 patients diagnosed with vascular abnormalities in adulthood (>16 years of age) was reported [11]. The authors analyzed data from the UK National Neurofibromatosis Service, encompassing 2068 adults (>16 years of age) with generalized NF1 from 2009 to 2019. Unfortunately, in the historical cohort, MRI brain imaging was only conducted in adults with NF1 when clinical indications were present (such as new or unexplained neurological symptoms or deficits, or in those undergoing surveillance for cerebral low-grade gliomas). Then, only a minority of patients underwent MRI, and only patients suspected of having vessel abnormalities on MRI were further investigated with MRA. In fact, routine MRA was not performed for all patients undergoing MRI. These limitations affect the estimated prevalence of cerebrovascular disorders, and they are probably underdiagnosed. A retrospective analysis of clinical records and cranial imaging results identified patients diagnosed in adulthood with various vascular abnormalities or complications, including arterial stenosis/occlusion, moyamoya arteriopathy, stroke (ischemic, hemorrhagic, or subarachnoid), aneurysms, other vascular malformations (arteriovenous malformations, fistulas), cavernomas, dissections, superficial siderosis, and venous thrombosis. The history of cranial radiotherapy was noted due to its association with vasculopathy, particularly moyamoya arteriopathy [26,53]. This cohort is the largest one that has been published until now and, despite the above-mentioned limitations, it is the most detailed one on cerebrovascular issues. Among the adult NF1 patients, 59.6% (1234/2068) underwent MRI cranial imaging, with a median age of 46 years (44% male; 56% female); 3.5% (43/1234) had a vascular abnormality. Notably, 60.5% (26/43) of these patients were symptomatic. Additionally, 0.65% (8) had more than one type of vascular abnormality. The main data are summarized in Table 1 and analyzed in the following paragraphs.

Among the 43 patients with vascular abnormalities, 40% (17/43) had radiological evidence of stroke, and 12/17 had at least one vascular risk factor, the most common ones being arterial hypertension and hypercholesterolemia. Notably, no patients with stroke had a clinical diagnosis of renal artery stenosis. Five patients (5/17, 29%) had stroke secondary to arterial stenosis/occlusion, with three out of five being symptomatic. Among those without large artery occlusion, seven were symptomatic (77%). Two patients were diagnosed with asymptomatic small cerebellar stroke, likely embolic, with no identifiable underlying cause. According to the neuroimaging stroke pattern, 52% were small-vessel, 35% were large-vessel, and 13% were watershed. Two patients experienced subarachnoid hemorrhage, both of whom had a history of arterial hypertension, and a saccular aneurysm was identified in one patient [11].

Overall, large vessel stenosis or occlusion was found in 18/43 (41.9%) patients. Thirteen patients had isolated arterial stenosis/occlusion without ischemic damage evident on MRI. Some 5/43 (12%) of patients exhibited moyamoya-type collateralization; however, only one had undergone preceding radiotherapy in childhood. Two symptomatic female patients (median age 33.5 years) presented with recurrent neurological events suggestive of cerebral ischemia and subsequently required superficial temporal artery to MCA and external carotid–internal carotid bypass surgeries, resulting in complete resolution of their symptoms at last follow-up (3 and 12 years, respectively). ICA was the most frequently involved artery (13/18, 72%), followed by MCA (7/18, 39%), anterior cerebral artery (ACA) (4/18, 22%), VA (3/18, 16%), and PCA (2/18, 11%). Stenosis was bilateral in only one patient, and seven patients had stenosis in more than one artery [11].

Some 10/43 (23%) of patients were diagnosed with an aneurysm, and 6/10 (60%) were symptomatic. Two patients had multiple aneurysms, and one had preexisting arterial hypertension. None had a family history of aneurysms. The most common aneurysm location was ICA (4), followed by MCA (2), posterior communicating artery (PCOM) (1), anterior choroidal artery (1), ACA (1), VA (1), internal mammary artery (1), thyrocervical trunk (1), and brachial artery (1) [11]. The thyrocervical trunk aneurysm was associated with an overlying plexiform neurofibroma, while no other aneurysms were linked to adjacent lesions or gliomas. Notably, one patient had seven aneurysms, six located in the left and right ICA and one in the ACA, associated with generalized ectasia of the cerebral arteries. This patient had a history of bypass surgery for aortic stenosis and renal transplants for renovascular disease, and no alternative diagnosis for the multiple vascular abnormalities was identified, although other genetic risk factors beyond NF1 may have been present. One patient in their mid-30s presented with sudden arm swelling and was subsequently diagnosed with a brachial artery aneurysm, requiring surgical intervention with a brachial–brachial anastomosis [54].

Incidental cavernous angiomas were found in 3/43 (6.9%) of patients; in two cases, these were late complications of radiotherapy for optic pathway glioma in childhood [11]. Furthermore, 3/43 (7%) patients (all males) experienced arterial dissections, with one occurring in the iliac artery. Two patients (one male, one female) were diagnosed with superficial siderosis after presenting with sensorineural hearing loss and spastic paraparesis, likely due to prior surgeries (brain surgery for a pilocytic astrocytoma in one and surgery for high cervical neurofibromas in the other). Lastly, a female patient in her early 60s presented with a neck mass that was later identified as an external jugular vein thrombosis, which was treated with antiplatelet therapy [11].

Finally, eight patients had multiple vascular abnormalities: two had an aneurysm and stroke; one had vessel ectasia, stenosis, and an aneurysm; one had a subarachnoid hemorrhage and dissection; one had a stroke, aneurysm, and stenosis; one had a stroke, stenosis, and cavernous angioma; one had a stenosis and vascular malformation; and one had a combination of stenosis, moyamoya collateralization, stroke, cavernous angioma, and aneurysm.

Finally, in a study of adult NF1 patients who had MRI cranial imaging, 3.5% (*n* = 43) had a vascular abnormality diagnosed in adulthood [11]. This is in keeping with previous studies of vasculopathy in NF1 [13,16,18,19]. The absence of longitudinal data does not allow us to determine whether these vascular abnormalities are congenital or developmental. Adult patients were mostly symptomatic for neurovascular abnormalities; 60% were symptomatic, although MRA was not routinely performed in all patients. Thus, the proposed rate may be an underestimation. Interestingly, in another cohort of patients with NF1 [11], the median age of onset of stroke was 44 years, compared to the national median of 77 years in the general UK population in the same years (National Institute for Health and Care Excellence, 2019). The majority of strokes in patients with NF1 were ischemic (76.4%) and were not directly related to arterial stenosis/occlusion. This contrasts with a previous study of hospitalized patients with NF1, where the odds of stroke diagnosis in adults were elevated compared to the general population, but this was primarily because of a significantly higher hemorrhagic stroke subtype [20]. Aneurysms were the third most common vascular abnormality in this cohort, but their rate was probably underestimated, as small aneurysms may be missed without routinely performing MRA.

The following table (Table 2) summarizes the main findings of the above described cohorts regarding the prevalence of cerebrovascular issues in NF1 patients.

One of the main findings is a wide range of prevalence of vascular lesions, and several reasons can account for this. One of them, as previously reported, is the lack of systematic screening in children and adults with NF1 because it is not recommended (not only for vascular issues but for parenchymal lesions too). In fact, only symptomatic patients underwent MRI in large cohorts, and only a minority of patients who underwent MRI were proposed for MRA too. Another limitation is the diagnostic performance of MRA, but for large arteries, false positives are more frequent than false negative findings. However, a cost-effective analysis was not performed to assess the effectiveness of systematic screening, and it is not possible to assume that the prevalence would significantly change in this case.

## 3. Focus on Intracranial Stenosis: Diagnosis and Management

Intracranial stenosis is among the most prevalent cerebrovascular hallmarks of NF1 and, in comparison with other vascular manifestations (e.g., aneurysms, vascular malformations, etc.), it is less likely to be incidental and not likely causally related to NF1 (not only in the pediatric age group). In fact, the mean age for stroke identified in the large cohort from UK [11] and the relatively scarcity of vascular risk factors support this interpretation versus other etiologies (e.g., atheromatic stenoses).

The primary concern regarding NF1-related cerebral vasculopathy is its likely underdiagnosis, as MRA is not routinely included in standard neuroradiological evaluations at baseline or during follow-up. Most of the reported studies are retrospective, with only a fraction of patients undergoing MRA, particularly when MRI did not indicate potential vascular involvement. A recent review highlighted that MRA is not part of the standard neuroradiological protocol for these patients [55]. In this same review, the incidence of cerebrovascular anomalies was reported at 1.3% [55]. An earlier case series from over a decade ago advocated for the inclusion of MRA in the standard neuroradiological assessment of children with NF1 [51]. This recommendation aligns with the guidelines from the National Institutes of Health (NIH) Consensus Development Conference [56], which stipulate that brain MRI should be performed only for children presenting with clinical indications such as headaches, visual deficiencies, orbitofacial masses, macrocephaly, brain neoplasms, developmental delays, pontine signs, suspected paramedian diencephalic syndrome, or when an entire deletion of the NF1 gene is detected [16,57]. Current recommendations remain focused on tumor screening and surveillance rather than vascular assessment [58]. In the aforementioned series [51], a total of 14 stenoses and/or occlusions of intracranial arteries were found in 6 patients: 5 involved the ICA, 3 the MCA, 3 the ACA, and 3 the PCA. Notably, only 7 of the 14 stenoses and/or occlusions identified by MRA were detectable via MRI.

**Figure 1:**
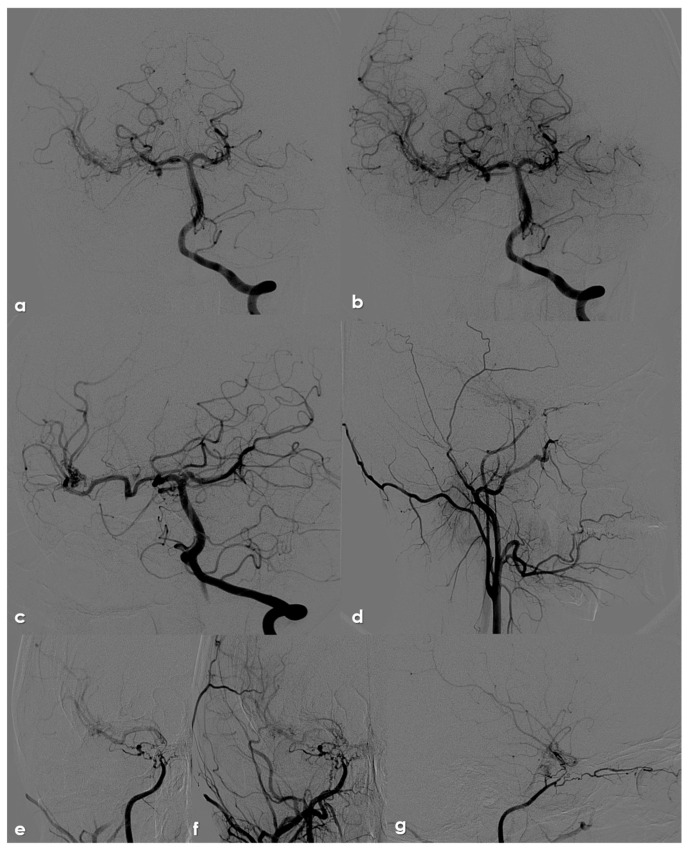
Digital subtraction angiography (DSA) of an 18-year-old patient with genetically confirmed NF1. Panels (**a**,**b**): posterior–anterior view from left VA injection in the early (**a**) and late (**b**) arterial phase, showing the right MCA supplied by the PCA through the posterior communicating artery and a tiny network of arterioles along the course of the distal M1 and M2 segments. Panel (**c**): left oblique view from left VA injection with a better visualization of the posterior communicating artery. Panel (**d**): lateral view from right common carotid artery injection, showing the absence of flow in the right ICA at the ophthalmic segment, the tiny caliper of the extracranial and intracranial course of ICA, and the network of vessels along the ICA terminus and the M1 MCA. The ophthalmic artery is supplied through external carotid artery branches (through the internal maxillary artery) and through an erratic network of arterioles arising from the intracranial ICA. Panels (**e**,**f**): posterior–anterior view (e) and magnification (**f**) from right common carotid artery injection, showing the network of arterioles across the ICA occlusion, reconstituting an alternative pathway to supply the MCA. Panel (**g**): lateral view of the same series, showing the interconnected network supplying the MCA and the ophthalmic artery.

**Figure 2:**
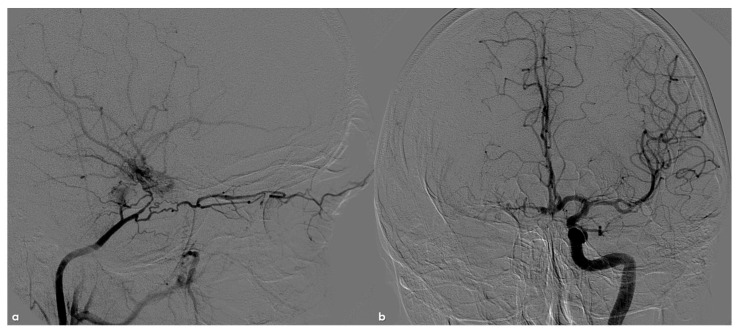
DSA in the same patient as in Figure 1. Panel (**a**): lateral view from right common carotid artery injection, showing two separated network of arterioles from the ICA, supplying the ophthalmic artery and the MCA. Panel (**b**): posterior–anterior view from left Ica injection, showing the minor contribution of the right ACA to the ipsilateral MCA supply through the anterior communicating artery (the posterior communicating artery pathway is prevalent).

**Figure 3:**
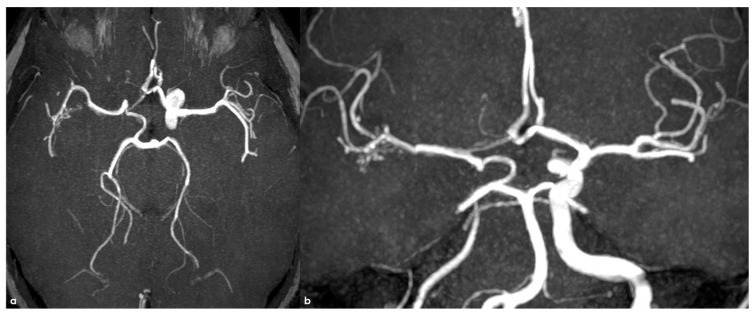
Time-of-flight intracranial MRA reconstructed using maximum-intensity projections/multiplanar reconstruction (MIP/MPR) protocol in the same patient as in Figure 1 and Figure 2. Panel (**a**): axial view showing the huge contribution of the right posterior communicating artery to the ipsilateral MCA supply and a tiny network of arterial vessels along the M2 MCA. Panel (**b**): Coronal view showing the same details from a different obliquation, confirming them.

A critical observation requiring clarification pertains to the etiology of intracranial stenoses in NF1 patients, especially concerning the differentiation between congenital and acquired pathologies. The lack of established guidelines for routine MRI and MRA studies in NF1 patients is a significant limitation in accurately determining the epidemiology and natural history of the disease, as well as defining a timeline for its progression. The documentation of intracranial stenoses in both pediatric and adult patients does not inherently imply a congenital origin. Specifically, in children, an etiological pathway similar to that of focal cerebral arteriopathies must be considered. While the hereditary condition may impact angiogenesis, it likely represents a “first hit,” necessitating a second event for the phenotypic expression of the arteriopathy [59]. Consequently, even when a diagnosis is made in children, it may reflect a multifactorial etiology, where genetic predisposition could justify early arterial pattern changes, blending congenital anomalies with acquired abnormalities from in utero development into the neonatal period [59]. This explanation could also account for the high proportion of asymptomatic incidental diagnoses reported in both childhood and adulthood across available studies [11,51]. Moreover, this hypothesis aligns well with cases involving both extra- and intracranial vascular pathologies, such as those affecting the ICA, as illustrated in Figure 1, Figure 2 and Figure 3.

The presence of an occluded arterial segment may be interpreted as either aplasia/regression or occlusion. Both scenarios can lead to significant collateralization, which may attempt to reconstruct the absent arterial segment [60,61]. The collateral or reconstitution pattern remains unchanged in cases of aplasia and early occlusion, qualifying similarly as an acquired arteriopathic process at a very young age, as described in clearly acquired cases [62]. Similarly, the dynamics of collateralization could be modifiable rather than simply representing embryological remnants, as previously noted for the MCA network or twig MCA [63,64,65,66,67,68,69]. Comparable considerations apply to moyamoya arteriopathy associated with NF1, where the proposed cases do not necessarily exhibit extensive collateral networks typical of moyamoya disease, as shown in MRA images presented by Currao et al. [70]. In instances where the collateral network is notably prominent on MRA [11,51,71], the MCA maintains its trajectory and branching, particularly in the M2 segments, raising the possibility of a distinct type of arteriopathy, such as twig MCA [63,64,65,66,67,68,69]. Most patients described in the literature underwent MRA, while digital subtraction angiography (DSA) findings are only available for a minority, typically as part of presurgical evaluations [72]. Furthermore, some well-documented cases in the literature likely represent moyamoya arteriopathy, and the embryological interpretation of moyamoya disease as a neurochristopathy is particularly intriguing, given the potential role of neurofibromin in neural crest development [73,74,75]. From this perspective, the understanding of central and peripheral nervous system tumors associated with NF1 is significantly clearer than that of vascular manifestations [71]. NF1 has yet to be classified among neurochristopathies from a vascular standpoint; however, its neurovascular and systemic involvements exhibit patterns similar to those described for ACTA2 mutations, PHACE syndrome, and RNF213 vasculopathy, including moyamoya disease [75].

In adults with NF1, a multifactorial genesis may also be considered, influenced by acquired factors such as hypertension. However, there is significantly less information regarding adults compared to children, and the natural history of intracranial arteriopathy in NF1 patients remains largely unknown. Similar to the etiopathogenesis, it is unclear whether a genotype–phenotype association exists or if there is a preferential association with other disease manifestations, such as optic nerve glioma. This uncertainty is compounded by selection bias based on the indications for neuroimaging studies, particularly in children and young adults.

The management of moyamoya arteriopathy in NF1 is not well defined. The neurosurgical and pediatric literature presents small, single-institution series [21,22,72]. Moyamoya disease is a progressive arteriopathy, yet little is understood about the natural history of moyamoya syndrome associated with NF1. Lin et al. [22] conducted a retrospective cohort study on moyamoya arteriopathy in NF1 patients, finding a radiological progression rate of 59% and a clinical progression rate of 44% over an average follow-up of about 5 years, with only 4 out of 34 (11.8%) patients experiencing a stroke. These findings support the proposal for both direct and indirect revascularization for these patients. A different cohort [72] presented similar data on a smaller sample, reinforcing the case for revascularization. The literature review by Lehman et al. [49] indicated that nearly all patients with NF1-related moyamoya arteriopathy underwent surgical treatment. In summary, the same treatment protocols proposed for patients with Moyamoya disease are currently recommended for NF1-related moyamoya syndrome [21,76,77,78,79,80,81,82,83,84]. Some data suggest that early intervention with arterial revascularization and pial synangiosis may reduce stroke risk in children with NF1 if implemented before the onset of symptoms [21]. Long-term studies of pediatric patients who received surgical revascularization and were followed into adulthood indicate a near elimination of recurrent ischemic strokes; however, the risk of de novo hemorrhage increases over time [84]. Nonetheless, the surgical treatment and consensus regarding the management of asymptomatic children with moyamoya remain contentious, particularly for those with NF1, and no strong evidence is available.

Even less is known about managing intracranial stenoses not associated with moyamoya arteriopathy in NF1 patients, whether they are children or adults. Specifically, it remains undefined whether antithrombotic therapy could be effective or what control methods and target levels should be established for key vascular risk factors, such as hypertension. For diagnoses made in adulthood, the possibility of an atheromatous etiology should not be disregarded, even if it is not always clearly demonstrable. In such cases, the management would align more closely with standard approaches used for intracranial atheromatous disease, regardless of hereditary conditions [85]. However, even in atherosclerotic stenosis, the usefulness of antiplatelets in primary prevention has not been demonstrated. Therefore, it cannot be recommended, but it has been suggested by expert consensus. Extrapolating from the above cited guidelines [85], in patients with severe symptomatic stenosis and hemodynamic compromise, induced arterial hypertension can be considered as a rescue treatment option in the acute phase, while in the chronic phase the target blood pressure is thought to be no different from in general patients (e.g., <130/80 mmHg). No clear suggestion can be provided about lipid-lowering therapy.

**Figure 4:**
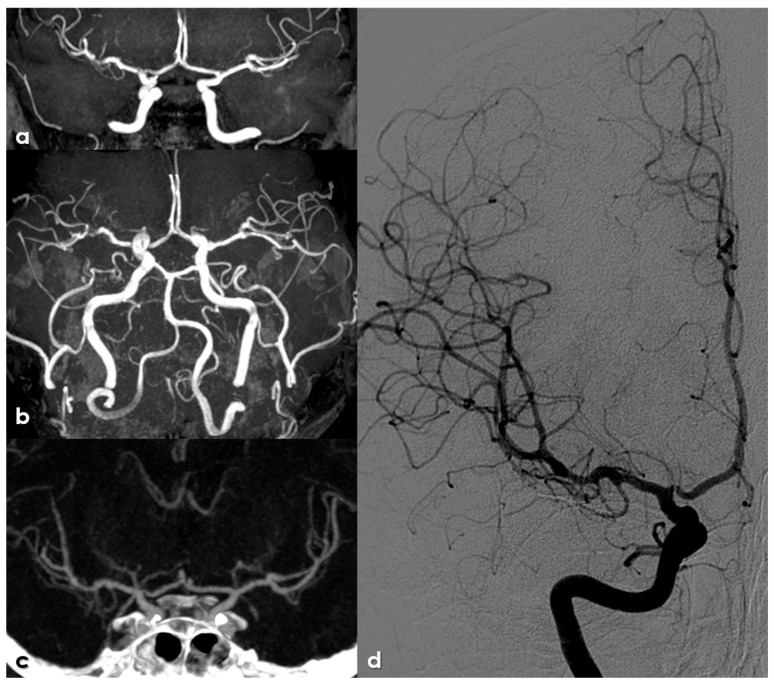
MRA, CTA and DSA of a 58-year-old woman with NF1. Panels (**a**,**b**): TOF MRA in coronal view with different obliquations, showing a flow gap in the right distal M1 MCA and in the left posterior M2 MCA branch, suggestive of stenosis. Panel (**c**): CTA (MIP/MPR protocol) in coronal view, showing very mild arterial stenosis in the same sites. Panel (**d**): DSA (posterior–anterior view from right ICA injection, showing a sequential mild stenosis in the right distal M1 MCA and a focal stenosis in the right A1 ACA.

**Figure 5:**
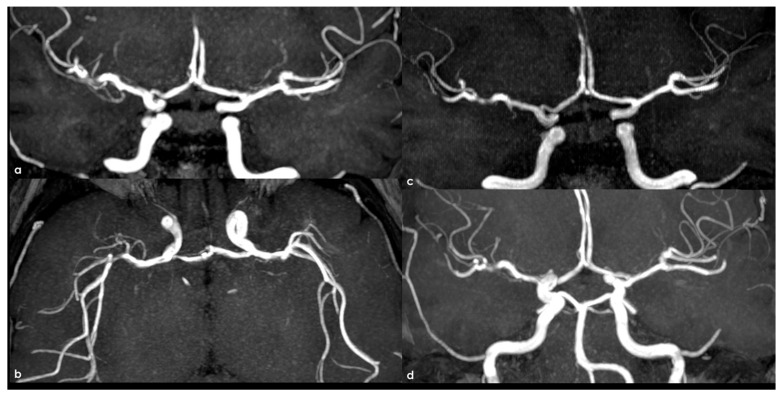
MRA in the follow-up in the same patient as in Figure 4 at 2 years (panels (**a**,**b**)), 4 years (panel (**c**)) and 6 years (panel (**d**)). Panels (**a**,**c**,**d**) are coronal MIP/MPR images and panel (**b**) is an oblique axial reconstruction. During follow-up, an evolution of the stenoses was not found.

Figure 4 and Figure 5 illustrate an example of intracranial stenosis in an adult patient with NF1.

Finally, the optimal or recommended screening and follow-up program for neurovascular involvement in NF1 patients is lacking both in children and in adults. In fact, neurovascular issues have not been systematically addressed in these patients, considering the international recommendations for screening and monitoring do not support the routine use of MRI and MRA. From a diagnostic standpoint, there are no specific indications for patients with NF1 that differ from what is usually done in the study of intracranial steno-occlusive disease in general. The need for parenchymal imaging related to the main manifestations of NF1 favors MRI-based techniques, which are in fact usually the first ones used (both MRI and MRA). The need for further vascular investigations, as in other patients, currently positions CTA as a complement to better hemodynamic definition. DSA remains the gold standard but, being more invasive, is reserved for the subgroup of patients undergoing selection for revascularization treatment.

## 4. Conclusions

This review on neurovascular issues in NF1 highlights several critical findings regarding intracranial stenoses and associated vascular complications.

1. Multifactorial Etiology: The etiology of intracranial stenoses in NF1 patients is complex and likely multifactorial, involving both congenital anomalies and acquired conditions such as hypertension. This complexity necessitates a deeper understanding of the genetic and environmental interactions that may influence vascular development.
2. Underdiagnosis of Vascular Abnormalities: There is a significant underdiagnosis of vascular complications, particularly intracranial stenoses, due to the lack of routine use of MRA in standard evaluations. The current diagnostic protocols primarily focus on tumor screening rather than comprehensive vascular assessment.
3. Epidemiological Insights: The prevalence of cerebrovascular abnormalities in NF1 varies between children and adults, with implications for clinical management. While pediatric patients show higher rates of stenotic lesions, adults often present with more diverse vascular complications, including strokes and aneurysms.
4. Clinical Management Challenges: The management strategies for NF1-associated vascular conditions, especially those not related to moyamoya arteriopathy, remain poorly defined. There is uncertainty regarding the efficacy of antithrombotic therapies and optimal control of vascular risk factors.
5. Need for Standardized Guidelines: The absence of standardized screening and follow-up protocols for neurovascular involvement in NF1 patients underscores the need for updated clinical guidelines. Such guidelines should incorporate regular neuroimaging to detect vascular abnormalities early, particularly as patients transition from pediatric to adult care.
6. Future Research Directions: There is a pressing need for longitudinal studies to better understand the natural history of intracranial arteriopathy in NF1, including the potential for early intervention to mitigate risks of stroke and other complications. In particular, a strategy of screening cerebrovascular manifestations starting in childhood should be considered, in order to assess reliable prevalence, to define clinical phenotypes and natural history, and to test different treatment strategies.

In summary, while the recognition of vascular issues in NF1 is improving, significant gaps in diagnosis, management, and research remain. Addressing these gaps through enhanced clinical protocols and research initiatives is essential for improving patient outcomes in this population.

## Figures and Tables

**Table 1:** Neurovascular manifestations in NF1 in a large national UK cohort (total 43) [11].

Category	Details
Stroke	17/43 (40%) Male to Female Ratio: 11:6 Median Age: 44 years (Range: 20–57 years) Ischemic Stroke: 13 patients (Range: 20–57 years) Subarachnoid Hemorrhage: 2 patients Hemorrhagic Stroke: 1 patient (hypertensive) Both Ischemic and Hemorrhagic Stroke: 1 patient Symptomatic Patients: 13 of 17 (76%; Male–Female 9:4) Stroke Secondary to Stenosis: 5 patients (29%; Male–Female 3:2) Stroke without Stenosis: 7 symptomatic (77%; Male–Female 6:1) Patients with Vascular Risk Factors: 12 of 17
Stenosis/Occlusion	18 patients Patients with Large Vessel Occlusion and Stroke: 5 Isolated Arterial Stenosis/Occlusion: 13 patients (Median Age: 37 years; Male–Female 4:9) Commonly Affected Arteries: Internal Carotid (72%), MCA (39%), ACA (22%), Vertebral (16%), PCA (11%)
Aneurysms	10 (23%; Male–Female 3:7; Median Age: 34 years) Symptomatic Aneurysms: 6 (60%; Median Age: 38 years; All Female) Common Aneurysm Sites: Internal Carotid (4), MCA (2)
Other Vascular Abnormalities	Vascular Malformations: 2 patients (5%; Female, mid-30s) Incidental Cavernomas: 3 patients (6.9%; Two post-radiotherapy) Arterial Dissections: 3 patients (7%; Median Age: 40 years) Superficial Siderosis: 2 patients (post-surgery complications) External Jugular Vein Thrombosis: 1 patient (treated with antiplatelets) Patients with Multiple Abnormalities: 8

**Table 2:** Summary of data about the prevalence of cerebrovascular issues in NF1 patients.

Reference	Number of Patients	Cohort Type	Diagnostic Technique	Country	Main Findings
[49,50]	398	Pediatric	MRI (78%), MRA (46%)	USA	Prevalence of cerebral vasculopathy: 4.8% (15/312); 50% asymptomatic; manifestations: moyamoya arteriopathy, stenosis/occlusion.
[51]	6	Pediatric	Not reported	Italy	Multiple intracranial stenoses/occlusions: 7.4% prevalence.
[52]	40	Pediatric	Transcranial Doppler, MRI	Brazil	Vascular changes: 7.5% (3/40); 2 symptomatic; cerebrovascular changes confirmed by MRA in 75%.
[11]	2068	Adult	MRI (59.6%), MRA (not routinely performed)	UK	Prevalence of vascular abnormalities: 3.5% (43/1234); 60.5% symptomatic; 40% had evidence of stroke (median age 44).

## Data Availability

No new data were produced in this paper.

## Abbreviations

ACA	anterior carotid artery
CTA	computed tomography angiography
DSA	digital subtraction angiography
ICA	internal carotid artery
MCA	middle cerebral artery
MRI	magnetic resonance imaging
MRA	magnetic resonance angiography
PCA	posterior cerebral artery
VA	vertebral artery
